# Vitamin D, pregnancy and caries in children in the INMA-Asturias birth cohort

**DOI:** 10.1186/s12887-021-02857-z

**Published:** 2021-09-03

**Authors:** Claudia Suárez-Calleja, Jaime Aza-Morera, Tania Iglesias-Cabo, Adonina Tardón

**Affiliations:** 1 Instituto Universitario de Oncología del Principado de Asturias (IUOPA), Instituto de Investigación Sanitaria del Principado de Asturias. ISPA. Universidad de Oviedo, Asturias, Spain; 2 SESPA (Servicio de Salud del Principado de Asturias), Asturias, Spain; 3 Unidad de Consultoría Estadística, Universidad de Oviedo, Asturias, Spain; 4 Instituto Universitario de Oncología del Principado de Asturias (IUOPA), Instituto de Investigación Sanitaria del Principado de Asturias. ISPA, CIBER de Epidemiología y Salud Pública. Universidad de Oviedo, Asturias, Spain

**Keywords:** Vitamin D, 25-hidroxivitamin D, 25(oh)d, Caries, Pregnancy, Childhood

## Abstract

**Background:**

Vitamin D is traditionally associated with the metabolism of calcium and phosphorus, a process essential for the mineralization of hard tissue such as bone or tooth. Deficiency of this vitamin is a problem worldwide, however. Given the possibly significant role of Vitamin D in odontogenesis in children, the objective of our study was to determine the influence of vitamin D levels in the blood on dental anomalies in children between 6 and 10 years of age, by means of 25-hydroxy vitamin D tests performed during pregnancy and the first years of life.

**Methods:**

The data analyzed were sourced from data belonging to the INMA-Asturias birth cohort, a prospective cohort study initiated in 2004 as part of the INMA Project. The 25-hydroxy vitamin D (25(OH)D) test was performed with samples from 188 children in the INMA-Asturias birth cohort with a dental examination performed between 6 and 10 years of age. The samples were taken at three stages: in the mother at 12 weeks of gestation, and subsequently in the child at 4 and 8 years of age. Diet, nutritional and oro-dental hygiene habits were also analyzed by means of questionnaires.

**Results:**

The results indicate a significant association between caries and correct or incorrect brushing technique. With incorrect brushing technique, the prevalence of caries was 48.89%, but this dropped to 22.38% with correct brushing technique. An association was also found between tooth decay and frequency of sugar intake. The prevalence of caries was 24.54% with occasional sugar intake, but this rose to 56% with regular sugar intake. On the other hand, levels < 20 ng/ml in both mother and child at 8 years of age would also be risk factors (OR_gest_ = 2.51(1.01–6.36) and OR_8years_ = 3.45(1.14–11.01)) for the presence of caries in children. The risk of caries practically tripled where 25(OH) D values were < 20 ng/ml.

**Conclusions:**

Although incorrect brushing technique and regular sugar consumption was found to be the main cause of caries in the children, the low concentrations of vitamin D in the blood of the pregnant mothers may have magnified this correlation, indicating that the monitoring of vitamin D levels during pregnancy should be included in antenatal programmes. It is particularly striking that 50% of the children were deficient in vitamin D at the age of 4, and that dental floss was practically absent from regular cleaning routines.

## Background

Vitamin D deficiency is a worldwide problem of epidemic proportions and a common metabolic condition [1–3]. Vitamin D is a fat-soluble vitamin involved in calcium and phosphorus metabolism, an essential process for the calcification of hard tissue and hence the mineralization of teeth and bones [4–6]. Tooth development or odontogenesis is a long process that begins around the 13th week of prenatal development and is usually completed in early adolescence. It is a dynamic process that involves the interaction of the structures under formation with the environment [7], hence the intrauterine environment, including the nutritional status of the mother, may affect the development, formation and mineralization of the child’s primary teeth [8].

Dental caries is a complex infectious disease arising from tooth-adherent bacteria that metabolize sugars to produce acid which then attacks dental tissue, resulting in pain, discomfort and problems chewing, and thus adversely affecting quality of life [9–11]. Among the most common chronic diseases in children and adults [12–14], dental caries are estimated to affect 60 to 90% of school-age children in industrialized countries, as well as many adults [15]. The multifactorial nature of the disease makes it difficult to isolate any single factor that causes caries to develop [9, 16–18], but a susceptible host, cariogenic diet and cariogenic bacteria, and their interaction over time, are the main factors to consider. Some authors argue that the most important factor for the development of caries is the dental biofilm composed of the community of bacteria that live in the oral cavity, and hence that the role of the other factors is merely to interact and modify the pathogenicity of these bacteria [16].

The quantification of caries is most often performed using the Decayed, Missing, and Filled teeth (DMFT) index, for over 50 years the benchmark and most commonly used tool worldwide for measuring the prevalence of decayed, missing and filled teeth. It is expressed as the total number of teeth that are decayed (D), missing (M), or filled (F) in an individual. The teeth not counted are unerupted teeth, congenitally missing teeth or supernumerary teeth, teeth removed for reasons other than dental caries, and primary teeth retained in the permanent dentition. This index is the most important index in epidemiological studies to determine the oral health of the population [19], and has been used to study the contribution of oral health services to changes in caries prevalence in the community [20].

The active form of vitamin D is vitamin D3 or cholecalciferol, and is mostly synthesized when the skin is exposed to ultraviolet radiation (UVB) from the sun; less than 10% of vitamin D is obtained from food [3, 21–23]. Food-derived vitamin D is absorbed in the small intestine, most effectively where fat is present. Slimming products that block fat metabolism and diseases of the small intestine may therefore affect vitamin D absorption [24]. Once in the blood, vitamin D metabolism begins in the liver, where it is converted to 25-hydroxy vitamin D (25(OH)D). This is the main serum form and the most reliable way to determine vitamin D levels as it is a measure of the total amount of vitamin D, whether obtained endo- or exogenously [25, 26].

Vitamin D deficiency has traditionally been associated with rickets in children, an anomaly in the mineralization of growing bone and cartilage. It is also frequently associated with variations in tooth morphology and oral anomalies such as periodontal and dental abscesses with no history of caries or trauma [22, 27] In adults, vitamin D deficiency is associated with osteomalacia, and exacerbates osteopenia, osteoporosis and fractures. Because of its anti-inflammatory and immune-modulating properties, vitamin D has also been linked to reduced morbidity in a number of diseases [28]. Several studies have found decreased mortality among dialysis patients receiving vitamin D supplements [29], while low levels of 25(OH) D in blood increased mortality in patients with chronic disease prior to dialysis [30]. There is also evidence that increased intake of vitamin D in children may decrease the risk of the development of type 1 diabetes mellitus [31], and some observational studies in human and animals support the argument that vitamin D has a positive function in cancer prevention and survival. This may be related to its role in the regulation of cell growth and differentiation [32, 33].

For all these reasons and given the possibly significant role of vitamin D in the development of dentition in children, we proposed to study the influence of vitamin D levels during pregnancy and the first years of life on dental anomalies in children between 6 and 10 years of age. Previously, our research group evaluated the concentration of 25(OH) D in children and mothers of the INMA-Asturias birth cohort and reported a significant deficiency in these children at 4 years of age and their mothers during pregnancy [34, 35]. We also assessed how the concentration of 25(OH) D in the mother influenced the development of children’s social competence [36]. The main reasons to explain this serious vitamin D deficiency in the INMA Asturias cohort are sun exposure and diet. Sun exposure is the main source of obtaining vitamin D, being the scarce solar exposure the main reason for its deficiency. Asturias is a region in northern Spain, and therefore we should keep in mind the less exposure to sunlight of our children, both for our latitude (43°) and for greater cloudiness. The seasonal variation of vitamin D is described, and it is consistent with our findings. Circulating vitamin D levels in pregnancy were significantly greater in the months of June to September when there is greater sun exposure. We have found a high prevalence of insufficiency/deficiency in pregnant women and children, especially between October to May. The second source of vitamin D is the diet. Intake recommendations during pregnancy varies from 5 μg/día (RNI: Recommended Nutrient Intake, WHO), 10μg/day (EAR: Estimated Average Requirements) y 15μg/day (RDA: Recommended Dietary Allowance). More than 50% of pregnant women in our study did not reach the 5μg/day of estimated intake of total vitamin D (food and supplements), and its majority did not reach 10μg/day, which might indicate a low intake of vitamin D. For 1- to 18-year-old children, current recommendations for dairy vitamin D intake are 15ud/day (AAP: American Association of Pediatrics, IOM: Institute of Medicine). In our study, none of the children would reach the intake of 15ud/day of vitamin D, and even 75% of children would not reach the 5ud/day. There is, therefore, an important deficit intake of vitamin D in children of our cohort. These levels are even lower than in their mothers [34, 35].

The purpose of this article is to determine the prevalence of caries and investigate the association between the parameters of the dental examination and the concentration of 25(OH) D in the mother during gestation, and in the child at 4 and 8 years of age, in a study population of 188 children from the INMA-Asturias birth cohort who underwent a dental examination between 6 and 10 years of age.

## Methods

### Study design and participants

The INMA-Asturias birth cohort is a prospective cohort study that began in 2004 with the recruitment of a total of 494 pregnant women between May 2004 and July 2007 in Avilés Health Area III, in the autonomous community of Asturias. INMA-Asturias is part of the INMA project (www.proyectoinma.org), a network of cohorts with a wide geographic coverage of Spain, the aim of which is to study the effects of environmental pollutants on prenatal and child development [37]. The pregnant women recruited for this project met the following inclusion criteria: (1) residence in one of the study areas; (2) minimum age of 16; (3) single pregnancy; (4) not having followed an assisted reproduction programme; (5) giving birth in the corresponding hospitals of reference; (6) having no communication issues [37].

Our study was populated with 188 children from the INMA-Asturias birth cohort with a dental examination performed between 6 and 10 years of age. A 25(OH) D measurement was recorded for 178 of the study population during the 12th week of their mother’s pregnancy, and for 138 of the children at 4 and 8 years of age. Data on 25(OH) D levels was recorded in all three groups – in the mother during pregnancy, and in the child at 4 and 8 years of age – for 108 of the total of 188 children with dental examination. For the remaining 80 children, 25(OH) D data was recorded in one or two of the three groups.

To obtain the dental data, a trained dentist (J.A.M.) reviewed the computerized clinical history of the review data obtained by the Oro-Dental Health Units of Health Area III from examinations of 188 children aged between 6 and 10 conducted in 2012. This dental examination was based on the DMFT index to assess the prevalence of decayed, missing, and filled teeth. Subjects with a DMFT score of 0 were considered free of caries. Incipient and non-cavity caries (white lesions in the enamel) and developmental defects of the enamel such as hypocalcification and hypoplasia were taken into account [6, 38]. The professionals in our study used WHO diagnostic and classification criteria, and conducted the examinations using a dental examination probe, inspection mirror and adequate lighting.

In addition, as part of the INMA project, questionnaires gathered information during pregnancy and childhood on socio-demographic characteristics (including cultural level, family income and employment), diet, dietary supplements, lifestyle, sun exposure and skin pigmentation, as well as oral hygiene and dietary habits. The following variables related to oral-dental hygiene were recorded: frequency of brushing (once, twice, three times a day, rarely); type of toothpaste used, which we classified according to the fluoride content (A: fluoride-free, B: 250–805 ppm, C: 961–1105 ppm, D: 1227–1500 ppm); use of dental floss; use of mouthwash; six-monthly fluoride tray application; frequency of sugar intake, which we classified as occasional or regular.

For the measurement of 25(OH) D levels, a single blood sample was taken from the mother at 12 weeks of gestation, and from the children at 4 and 8 years of age. The samples were processed immediately and stored at − 70 to − 80 °C until analyzed. Quantification of maternal plasma 25(OH)D3 concentrations were obtained by the high performance liquid chromatography method, using a BioRAD kit in compliance with NCCLS (Clinical And Laboratory Standards Institute) protocols. The detection limit was 5 ng/ml; the interassay coefficient of variation was 4.5%. The assay was validated using German external quality assessment programs (DGKL-RfB_Referenzinstituc fur Bioanalytik), with satisfactory results in 100% of all cases [35]. In view of the continuing controversy regarding the optimal plasma levels for 25(OH) D, we classified these into three categories according to the recommendations of the Endocrine Society (2011), which appear to be the most widely used: deficient below 20 ng/ml (50 nmol/L), deficient between 20 and 29 ng/ml (50–74 nmol/L), and deficient above 30 ng/ml (75 nmol/L) [34–36, 39–41].

### Statistical analysis

A descriptive analysis was performed, providing absolute and relative frequency distributions for qualitative variables, and position and dispersion measures for quantitative variables. The relationships between qualitative variables were assessed with Pearson’s Chi-square test or Fisher’s test, depending on whether or not the hypothesis of expected frequencies was fulfilled. These relationships were quantified by crude odds ratios, and subsequently adjusted for confounding variables using multivariate logistic regression models. The significance level used was 0.05; statistical analysis was performed with R, version 3.6.0.

## Results

The mean 25(OH) D values recorded in the mother during pregnancy, and in the child at 4 and 8 years of age, were 28.03, 20.70 and 26.74, respectively (Table 1).

**Table 1:** Summary of quantitative variables

				Percentiles (%)		
	n	Mean	DT	0	50	100
25(OH) D pregnancy	178	28.03	11.18	6.40	26.90	66.90
25(OH) D 4 years	138	20.70	7.59	2.77	19.84	42.37
25(OH) D 8 years	138	26.74	9.05	9.00	26.10	53.00

**Fig. 1:**
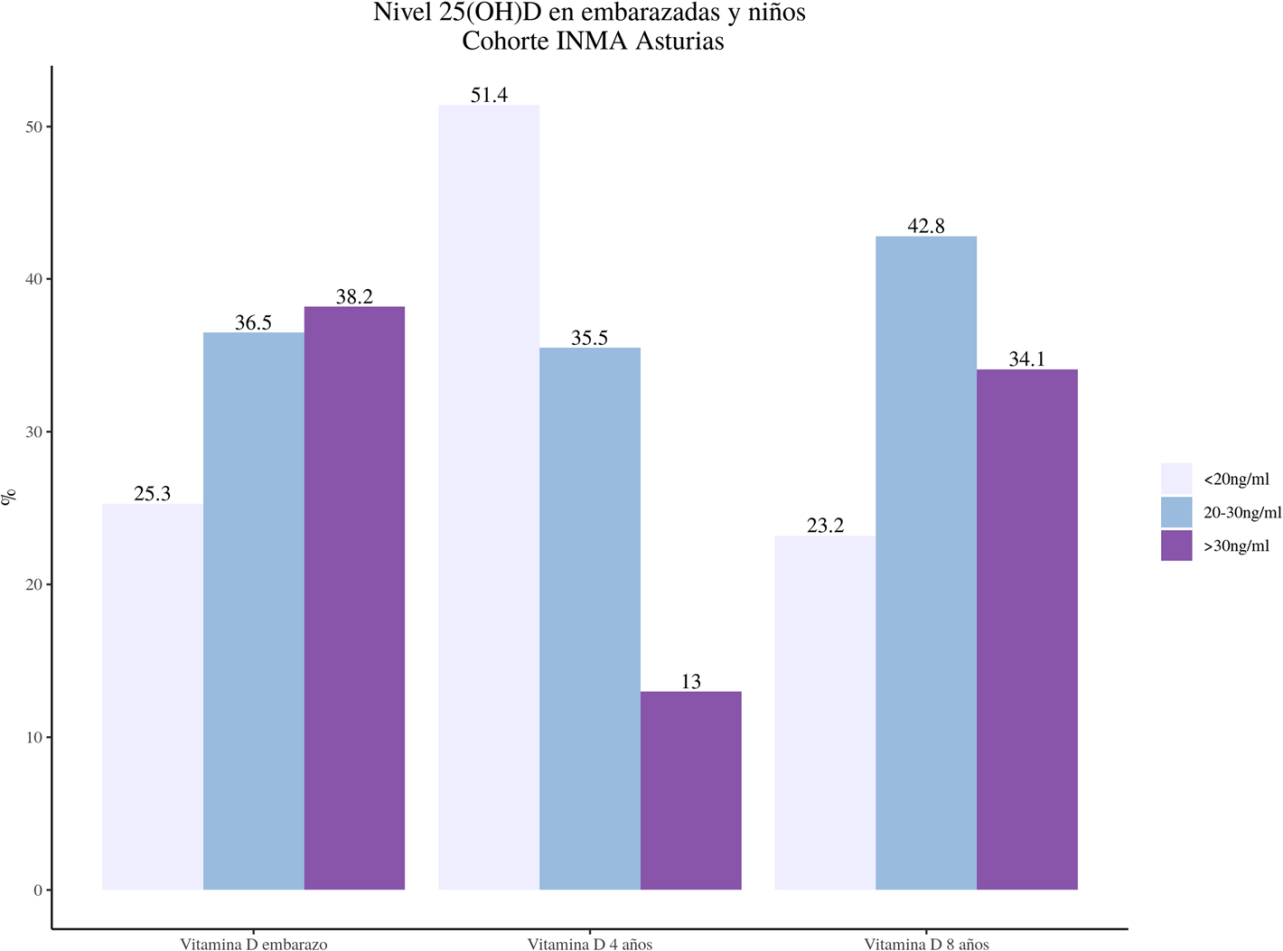
Frequency distribution in percentage of 25(OH) D in pregnant women and children of the INMA-Asturias birth cohort

In the mother at 12 weeks of gestation, the following frequency distribution was obtained: 25.3% presented plasma 25(OH) D values below 20 ng/ml, i.e., deficient; 36.5% presented values between 20 and 30 ng/ml, i.e., insufficient; and 38.2% presented values above 30 ng/ml, i.e., sufficient. In the child at 4 years of age, the following frequency distribution of plasma levels of 25OHD was obtained: 51.4% presented values below 20 ng/ml, 35.51% between 20 and 30 ng/ml, and 13% had sufficient levels above 30 ng/ml. The next 25(OH) D blood level measurement was performed at 8 years of age in the child: 23.2% presented deficient values of 25(OH) D, < 20 ng/ml; 42.8% presented values between 20 and 30 ng/ml; and 34.1% presented values > 30 ng/ml (Fig. 1).

As we can see in Fig. 1, almost 30% of the pregnant women (25.3%) presented with 25(OH) D deficiency at the beginning of their pregnancy. The children at ages 4 and 8 maintained this deficit, which rose as high as 51.4% at 4 years of age. With regard to caries, of the 188 subjects of our study (children with dental examinations carried out between 6 and 10 years of age), 71.3% were free of caries, while 28.7% had caries lesions. We analyzed the following variables related to oro-dental hygiene and eating habits: frequency of brushing; type of toothpaste according to fluoride content; use of dental floss; correct or incorrect brushing technique; frequency of sugar intake; six-monthly fluoride tray application; use of mouthwash. (Table 2).

**Table 2:** Variables of oro-dental hygiene and dietary habits

CARIES							
	Total		With caries		Without caries		***p*** value
	n	%	n	%	n	%	
**Frequency of brushing**							
1x/day or rarely	17	9	4	25.53	13	76.425	0.786
2x/day	103	54.8	32	31.07	71	68.93	
3x/day	68	36.2	18	26.47	50	73.53	
**Type of toothpaste**							
Fluoride free	8	4.3	2	25	6	75	0.899
250–805 ppm	147	78.2	44	29.93	103	70.07	
961–1105 ppm	33	17.5	8	24.24	25	75.76	
**Use of dental floss**							
Yes	1	0.5					
No	187	99.5					
**Brushing technique**							
Correct	143	77.06	32	22.38	111	77.62	**0.001**
Incorrect	45	23.93	22	48.89	23	51.11	
**Frequency of sugar intake**							
Occasional	163	86.7	40	24.45	123	75.46	**0.001**
Regular	25	13.3	14	56	11	44	
**Six-monthly fluoride tray application**							
Yes	11	5.9	4	36.36	7	63.64	0.515
No	177	94.1	56	28.25	127	71.75	
**Mouthwash**							
Yes	86	54.3	28	32.56	58	67.44	0.286
No	102	45.75	26	25.49	76	74.51	

With regard to the frequency of brushing variable, 9% reported brushing their teeth “almost never” or once a day, while 54.8 and 36.2% reported brushing twice or three times a day, respectively. We obtained the following results in relation to toothpaste used: 4.3% used type A toothpaste (fluoride free), 78.2% used type B (250–805 ppm), 17% used type C (961–1105 ppm), and 0.5% used type D (1227–1500 ppm). With regard to the dental floss variable, we found that only 0.5% of the children reported using dental floss routinely as part of their dental hygiene, while the vast majority (99.5%) did not. In relation to brushing technique, 77.06% of the children employed a correct brushing technique, while 23.93% did not. The frequency of sugar intake variable showed that 86.7% of the children consumed sugar only occasionally, while 13.3% did so regularly. Only 5.9% reported receiving a six-monthly fluoride tray application, compared to 94.1% who did not. As for the mouthwash variable, 54.3% reported using mouthwash regularly as part of their dental hygiene, compared to 45.75% who did not. Studying the relationship between these variables in the questionnaire and the appearance of caries, we found an association between caries and correct or incorrect brushing technique (*p* = 0.001 from Pearson’s Chi-square test). With incorrect brushing technique, the prevalence of caries was 48.89%, but this dropped to 22.38% with correct brushing technique. We also found an association between caries and frequency of sugar intake (*p* value = .002 from Fisher’s test). The prevalence of caries was 24.54% with occasional sugar intake, rising to 56% with regular sugar intake. The risk of caries while employing an incorrect brushing technique and consuming sugar on a regular basis was three times higher than for those who employed a correct brushing technique and consumed sugar only occasionally (OR_brushing_ = 3.53 (1.70–7.43) and OR_sugar_ = 3.62(1.49–9.01)). Adjusting these variables for 25(OH) D levels in the mother during pregnancy, we found that the risk of caries remained the same and even increased somewhat (Table 3).

**Table 3:** Association between caries and correct or incorrect brushing technique, and between caries and occasional or regular sugar intake, adjusting for 25(OH) D levels in the mother during pregnancy

**Association between caries and brushing technique in children of the INMA-Asturias birth cohort**			
	n	Unadjusted OR	Adjusted OR
Correct	143	Reference	
Incorrect	45	3.32 (1.64–6.75)	**3.53 (1.70–7.43)**
**Association between caries and brushing technique in children of the INMA-Asturias birth cohort**			
	n	Unadjusted OR	Adjusted OR
Occasional sugar	163	Reference	
Regular sugar	25	3.91 (1.65–9.50)	**3.62 (1.49–9.01)**

We also analyzed the possible association between 25(OH) D (both in the mother during pregnancy, and in the child at 4 and 8 years of age) and the development of caries. In Table 4, we assess the presence of caries as a function of 25(OH) D levels, first using the unadjusted odds ratio (OR), and then adjusting for the brushing technique and frequency of sugar intake variables. We found that levels < 20 ng/ml in both the mother during pregnancy and the child at 8 years of age would also be risk factors (OR_gest_ = 2.51(1.01–6.36) and OR_8years_ = 3.45(1.14–11.01 for the appearance of caries. The risk of caries practically triples where 25(OH) D values are < 20 ng/ml. However, no statistically significant association was found between serum 25(OH) D concentrations in children at 4 years of age and the development of caries.

**Table 4:** Association between 25(OH) D levels and caries adjusted for brushing technique and frequency of sugar intake variables

	Without caries	With caries	*p* value	Unadjusted OR	Adjusted OR
**25(OH) D during pregnancy**			0.029		
> 30 ng/ml	55 (43.31%)	13 (25.49%)		Reference	Reference
20 ng/ml	46 (36.22%)	19 (37.25%)		1.75 (0.79–3.99)	1.57 (0.66–3.77)
< 20 ng/ml	26 (20.47%)	19 (37.25%)		3.09 (1.34–7.35)	2.51 (1.01–6.36)
**25(OH) D at 4 years**			0.319		
> 30 ng/ml	11 (11.00%)	7 (18.42%)		Reference	Reference
20 ng/ml	34 (34.00%)	15 (39.47%)		0.69 (0.23–2.20)	0.42 (0.12–1.46)
< 20 ng/ml	55 (55.00%)	16 (42.11%)		0.46 (0.15–1.42)	0.35 (0.11–1.18)
**25(OH) D at 8 years**			0.177		
> 30 ng/ml	37 (37.76%)	10 (25.00%)		Reference	Reference
20 ng/ml	42 (42.86%)	17 (42.50%)		1.50 (0.62–3.78)	1.94 (0.71–5.64)
< 20 ng/ml	19 (19.39%)	13 (32.50%)		2.53 (0.95–6.99)	3.45 (1.14–11.01)

## Discussion

Our study revealed a high prevalence of pregnant women and children that presented with deficient levels of 25(OH)D. We were particularly struck by the fact that 51.4% of the children were vitamin D deficient at the age of 4. This deficit was consistent from the prenatal stage through to the child at 8 years of age, with mean 25(OH) D levels of 28.03, 20.70 and 26.74, in pregnancy and at 4 and 8 years of age, respectively, with vitamin D levels > 30 ng/ml deemed sufficient. Our main objective in this study was to determine the association between the dental status of children between 6 and 10 years of age from the INMA-Asturias birth cohort and serum 25(OH) D concentrations in the mother during pregnancy, and in the child at 4 and 8 years of age. The results support an inverse relationship between caries and serum 25(OH) D levels: we found that the risk of caries in the 6 to 10-year-old child practically tripled where 25(OH) D values were < 20 ng/ml in both the pregnant mother and the child at 8 years of age. It is also worthy of note that, within the dental hygiene parameters evaluated, incorrect brushing technique and regular sugar intake tripled the risk of developing caries.

The prevalence of caries in the 188 children in our cohort aged 6 to 10 years was 28.7%, while 71.3% were free of caries. In relation to oro-dental hygiene, we found an association between caries and brushing technique (Pearson’s Chi-square test, *p* = 0.001), and between caries and sugar intake (Fisher’s test, *p* = 0.002). Among the subjects who reported incorrect brushing technique, 48.89% had caries, compared to 22.38% in the children who reported brushing their teeth correctly.

One of the pillars of caries prevention, correct brushing technique assists the removal and destruction of dental biofilm, thus reducing the number of bacteria. If brushing is always performed correctly, the communities of microorganisms responsible for caries are prevented from recovering, and a satisfactory level of oral hygiene is maintained [42, 43].

Other studies have attributed the success of brushing to the use of toothpaste containing fluoride [12, 44, 45] or, preferably, fluoride combined with triclosan [46]. With the toothpaste, as we have already mentioned, the act of brushing itself breaks up the colonies of microorganisms and reduces the number of bacteria, but the fluoride provided by the toothpaste plays an essential role in helping the enamel to remineralize. In our study, we found no association between the type of toothpaste used and the development of caries. Our findings agree with a 2016 study conducted in Korea, which argued that the amount of fluoride found in toothpaste and mouthwashes would not be sufficient to maintain anti-acid activity in the mouth, regardless of the amount of fluoride they contained [47].

Among the children who consumed sugar occasionally, only 24.54% presented with caries, compared to 61.90% of children who reported regular sugar intake. The consensus among the studies reviewed is that a direct relationship exists between regular sugar intake and the development of caries [43, 45, 48], since sugars consumed in the diet are converted into acids by the fermentation action of the microorganisms in the oral cavity. Children must therefore brush their teeth with a soft brush from the moment the first teeth erupt in the oral cavity, and their sugar intake must be limited; these are the pillars of caries prevention in children.

Another factor studied in relation to the development of caries is vitamin D, which plays a fundamental role in the metabolism of calcium and phosphorus, essential for the calcification of hard tissues such as bones and teeth. A vitamin D deficiency during the intrauterine stage is believed to lead to ameloblast damage and hence enamel hypoplasia, the most common developmental anomaly in the formation of tooth enamel [4]. In our study, we analyzed the presence of caries in children according to 25(OH) D levels in the mother during pregnancy (at 12 weeks of gestation), and in the child at 4 and 8 years of age, adjusting for the oro-dental hygiene and dietary habits variables considered. We found that deficient levels of 25(OH) D, that is, < 20 ng/ml in the mother during pregnancy and in the child at 8 years of age would be risk factors (OR_gest_ = 2.51(1.01–6.36) and OR_8years_ = 3.45(1.14–11.01)) for the appearance of caries, almost tripling the risk of caries in the child between 6 and 10 years of age. No statistically significant association was found between serum 25(OH) D levels at 4 years of age and the development of caries.

A number of studies conducted in different geographical locations concur with our findings in associating a deficient level of vitamin D with increased risk of caries. A recent prospective cohort study of 1210 mother-child pairs in Japan associated a higher intake of vitamin D during pregnancy with a lower risk of dental caries in children aged between 3 and 4. However, the study relied on general questionnaires, habits and diet, rather than on vitamin D concentrations measured directly from blood samples [10]. In 2018, an inverse association was found between 25(OH) D levels and caries in a population of 6-year-old Swedish children supplemented with either vitamin D or a placebo for 3 months [49]. In Canada, preschool children with early caries were found to have lower serum 25(OH) D levels than the control subjects without caries [6]. Low levels of 25(OH) D during gestation have also been associated with increased risk of caries during the first year of life [5].

The direct comparison of results from around the world is complicated by differences among populations, and different geographical locations and study designs. In addition, many of the studies found in the literature regarding vitamin D and dental caries were conducted during World War I and World War II, since when health conditions, nutrition, and lifestyles have changed significantly [50]. Recent epidemiological studies on the association between vitamin D and caries are scarce [4–6, 10, 11, 27, 51–60], and we found none in Spain. Most of these studies, like ours, in addition to questionnaires, used serum tests to determine 25(OH) D levels [5, 6, 11, 49, 54–56, 58, 61, 62], the most reliable means of measurement. Some studies, however, used only a dietary questionnaire to estimate intake [10]; we consider this a constraint to reliably determining actual levels of vitamin D in the body. These studies relied on parents’ reporting of their child’s or the mother’s diet during pregnancy, without any direct measurement of serum vitamin D levels. As we have previously reported, the children of the INMA-Asturias birth cohort were characterized by a significant vitamin D deficiency in the mother during pregnancy [35], and in the child at 4 years of age [34]. Our study results indicated that vitamin D levels in the child at 8 years were consistent with prenatal levels in the mother. During pregnancy, 25.3% of the mothers had 25(OH) D values < 20 ng/ml, that is, vitamin D deficient; in the child at 4 years of age, this percentage had almost doubled, with 51.4% of children showing 25(OH) D deficiency; then at 8 years of age, the deficiency returned to similar values to those of the mother during the pregnancy, with 23.2% of the children presenting a deficiency. We have not found a clear reason why the age-dependant increase happens, but it may be hypothesized that the body surface area exposed to the sun increases as the infant become older. In a previous study in our region, Asturias [63], it was also found that serum 25OHD concentrations increase spontaneously with age in infants not receiving vitamin D prophylaxis, a finding in agreement with former studies [64, 65]. We believe it is of fundamental importance to provide mothers with nutritional education that promotes a diet rich in vitamin D, especially during the winter months; and, above all, to encourage outdoor lifestyles that allow the necessary amount of sun exposure, according to parameters such as latitude or time of day, from pregnancy through childhood. Vitamin D supplementation for children at risk is another option for consideration, though supplementation appears to be less effective than the effect of sunlight on the skin. A recent study demonstrated that vitamin D obtained from exposure to sunlight had a more positive effect than a vitamin D supplement on bone structure and certain hormones and minerals in vitamin D deficient rats, who presented with improved trabecular thickness, number and spacing after 10 days of sun exposure [66].

## Conclusions

1. We found a significant inverse association between caries in the child on the one hand, and 25(OH) D levels in the mother during gestation, and in the child at 8 years of age, on the other. These results are not consistent, as we were unable to observe a similar relationship with 25(OH) D levels in the child at 4 years of age.
2. The incidence of caries is related to brushing technique and frequency of sugar intake.
3. When we adjust for the brushing technique and frequency of sugar intake variables, we can observe that the concentration of 25(OH) D in the mother during pregnancy and later in the child influences the incidence of caries from 6 to 10 years of age.
4. The prevalence of pregnant women and children with deficient levels of 25(OH) D is high. This deficiency in the child at 8 years of age was consistent with that of the mother during pregnancy.
5. There is a need to promote outdoor lifestyles for mothers and children, from pregnancy and throughout childhood, to increase sun exposure for more efficient synthesis of vitamin D according to the geographical characteristics of the location; and to incorporate nutritional education into antenatal programmes.
6. Vitamin D supplementation, for pregnant women as well as children, is an option that should be given due consideration in cases of very advanced caries.

## Data Availability

The datasets used and/or analysed during the current study are available from the corresponding author on reasonable request.

## Abbreviation

25(OH)D: 25-hydroxy vitamin D
